# Shrinkage stress and elastic modulus assessment of bulk-fill composites

**DOI:** 10.1590/1678-7757-2018-0132

**Published:** 2019-01-07

**Authors:** Fabio Antonio Piola Rizzante, Rafael Francisco Lia Mondelli, Adilson Yoshio Furuse, Ana Flávia Sanches Borges, Gustavo Mendonça, Sérgio Kiyoshi Ishikiriama

**Affiliations:** 1 Case Western Reserve University, School of Dental Medicine, Department of Comprehensive Care, Cleveland, Ohio, USA; 2 University of São Paulo, Faculdade de Odontologia de Bauru, Departamento de Dentística, Endodontia e Materiais Odontológicos, Bauru, São Paulo, Brazil; 3 University of Michigan, School of Dentistry, Department of Biologic and Material Sciences - Division of Prosthodontics, Ann Arbor, Michigan, USA

**Keywords:** Resin composites, Polymerization stress

## Abstract

Bulk-fill composites were introduced in dentistry to accelerate clinical procedures while providing adequate outcomes. Concerns regarding the use of bigger composite increments rely on the polymerization shrinkage and shrinkage stress, which may generate gaps on the adhesive interface and result in a reduced success rate. Objective: To evaluate the polymerization shrinkage stress of different bulk-fill resin composites and their elastic modulus. Materials and Methods: Fourteen specimens were made for each of the nine different resin composites (seven with 12 mm^3^ and seven with 24 mm^3^): Surefill SDR flow (SDR), X-tra Base (XB), Filtek Bulk Fill Flowable (FBF), Filtek Z350XT Flow (Z3F); Tetric Evo Ceram Bulk Fill (TBF), X-tra Fil (XF), Filtek Bulk Fill (FBP), Admira Xtra Fusion (ADM) and Filtek Z350 XT (Z3XT). Linear shrinkage stress was evaluated for 300 s with the aid of a linear shrinkage device adapted to a Universal Testing Machine. For each composite group, seven additional specimens (2x2x25 mm) were made and Young's modulus was evaluated with a 3-point bending device adapted in a Universal Testing Machine with 0.5 mm/min crosshead speed and 50 KgF loading cell. Results: For 12 mm^3^ specimens, three-way ANOVA showed that only SDR and TBF generated lower stress after 20 s. Considering 300 s, TBF, SDR, and XF generated the lowest stress, followed by ADM, FBP, XB, and FBF, which were similar to Z3XT. Z3F generated the highest stress values for all time points. Considering 24 mm^3^ specimens after 20 s, all bulk fill composites generated lower stress than Z3XT, except XB. After 300 s, SDR, FBP, and ADM generated the lowest stress, followed by TBF and XF. For elastic modulus, one-way ANOVA showed that FBF, SDR, Z3F, and ADM presented the lowest values, followed by XB and TBF. FBP, Z3XT, and XF presented the highest elastic modulus among the evaluated composites. Conclusions: Bulk-fill resin composites presented equal to lower shrinkage stress generation when compared to conventional composites, especially when bigger increments were evaluated. Bulk-fill composites showed a wide range of elastic modulus values, but usually similar to “regular” composites.

## Introduction

Despite advances in adhesive dentistry, resin composites still tend to fail in extensive posterior restorations due to wear, medium to long term adhesive interface deterioration, technical sensitivity, polymerization shrinkage and inadequate polymerization, particularly in class II restorations with cervical margins located in dentin or cementum.^1^ ^–^ ^5^ Defects on the adhesive interface are generated by the characteristics of resinous materials during the polymerization process. Composites generate shrinkage (polymerization shrinkage) that depends on the material composition and volume.^5^ ^–^ ^10^ Shrinkage can generate stresses that may lead to the formation of micro gaps and, thus resulting in microleakage of saliva and bacteria, adhesive interface degradation, secondary caries, pulpal changes, and consequently, clinical failure of the restoration.^4^ ^,^ ^5^ ^,^ ^11^

The incremental insertion technique is recommended to ensure a better marginal integrity because it reduces the development of polymerization shrinkage stress.^12^ ^–^ ^14^ However, despite the advantages of the incremental technique in ensuring a better polymerization and stress distribution, this technique is more laborious, technically sensible and time-consuming.^8^ ^,^ ^10^ ^,^ ^15^

Bulk-fill resin composites are advised to be used in larger increments without compromising the degree of conversion (up to 4 mm according to some manufacturers). Concerns with the polymerization of large increments relies on the polymerization shrinkage and on the stresses generated in the tooth/restoration interface.^10^ ^,^ ^16^ ^–^ ^18^ Promising results have been reported with these materials, mainly due to lower polymerization shrinkage,^5^ ^,^ ^18^ ^,^ ^19^ which also depends on the composite organic/inorganic matrix composition and properties such as viscosity and elastic modulus.

Although several materials with different viscosities and handling characteristics are commonly classified as bulk-fill resin composites, their properties can change considerably, especially due to modifications in the organic matrix, with the incorporation of monomers with higher molecular weight, as well as changes in filler content and incorporation of stress relievers.^5^ ^,^ ^10^ ^,^ ^16^ ^,^ ^18^ ^,^ ^20^ ^–^ ^23^

Composites can be subdivided according to their consistency in low- and high-viscosity. Higher shrinkage stress for flowable composites are expected since they generally have a higher organic content when compared to microhybrid and nanoparticulate composites, which can result in greater polymerization shrinkage and lower mechanical properties.^22^ ^,^ ^24^ Similarly, a lower Young's modulus may allow stress dissipation during the polymerization process, thus reducing the stress when bigger increments are used.^25^ ^,^ ^26^ Given this discussion, the viscoelastic behavior (and its development during the polymerization process) and the volumetric shrinkage are critical during the generation of polymerization stress, showing the importance of stress development among composites with different viscosities.^25^ ^,^ ^26^ The hypothesis of this study was that the properties of bulk-fill and regular composites would be different. Thus, the objective was to evaluate the polymerization shrinkage stress and the elastic modulus of different bulk-fill resin composites.

## Materials and methods

**Figure 1:**
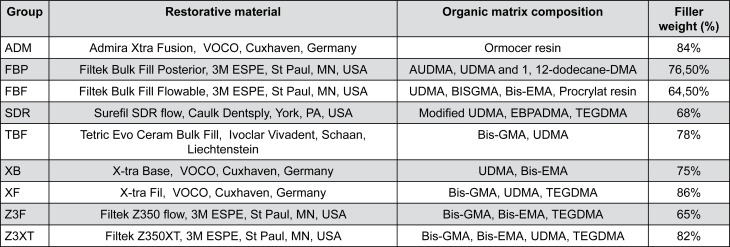
Different groups with respective composition and manufacturer

This study evaluated nine different resin composites (Figure 1), having as response variables: linear shrinkage stress (considering two levels of specimen volume: 12 mm^3^ and 24 mm^3^), and Young's modulus.

### Linear shrinkage stress

**Figure 2:**
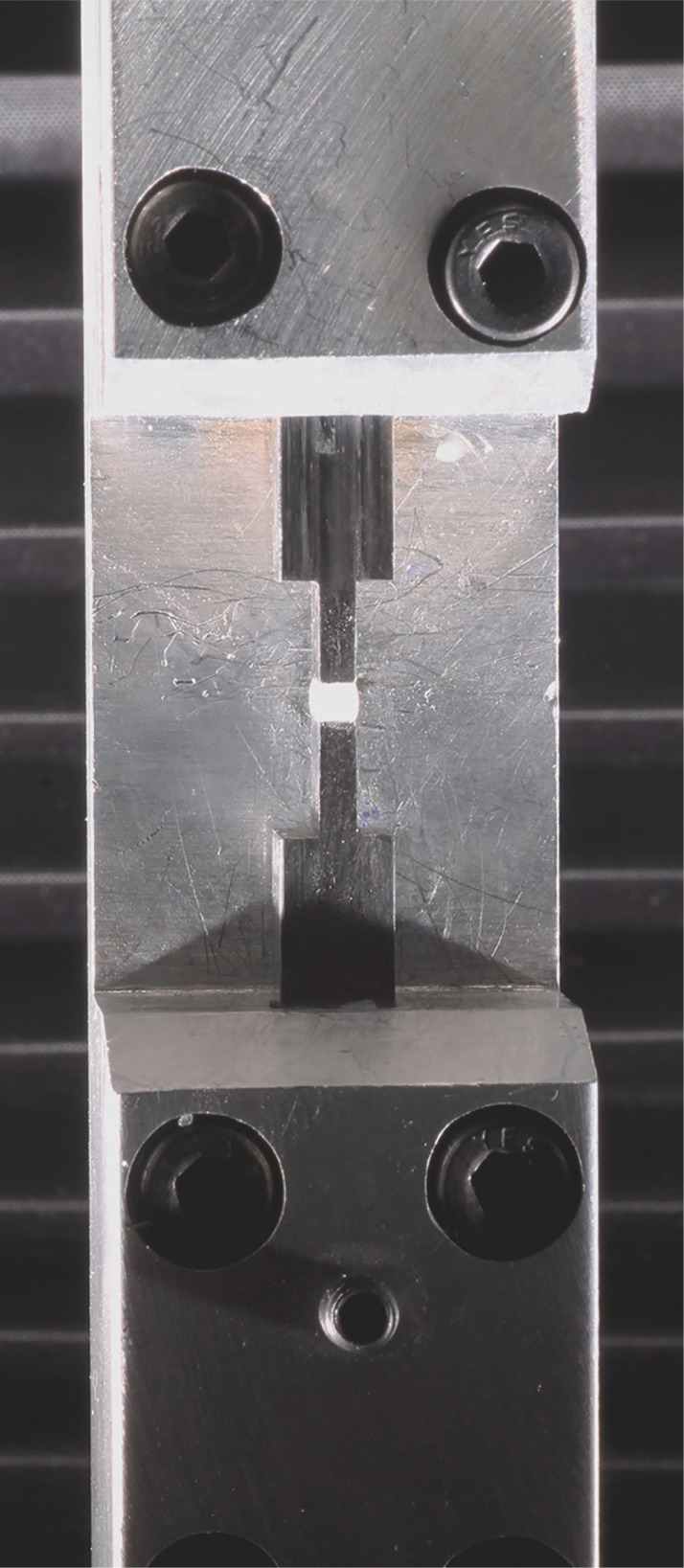
Universal Testing Machine (INSTRON) and metallic bases with composite specimen in position

The tensile stress test was used to evaluate the linear polymerization stress of the composites.^7^ ^,^ ^27^ For this test, seven 12 mm^3^ and seven 24 mm^3^ specimens of each resin composite were used. The restorative materials were inserted between two metallic bases with 6x2 mm surface dimensions (Figure 2). These bases were previously sandblasted (surfaces in contact with composites) with aluminum oxide, avoiding the need of applying an adhesive system.^20^

The metallic bases were adapted on a Universal Testing Machine (Instron model 3342, Instron, Norwood, MA, USA) by using an articulated arm connected to the 50 KgF load cell (upper base), and by using a BENCOR multi testing device (lower base) (Figure 2). This ensemble was used to perform a real-time evaluation of the forces generated during polymerization for 300 s.

To standardize the material volume, the resin composite was inserted between the bases, with 1 mm between them, resulting in a 12 mm^3^ constant volume and a 1.5 C-factor. The same test was repeated with 2 mm between the bases, resulting in a volume of 24 mm^3^ and a reduced 0.75 C-factor due to the increase in the height between bases. Light curing was performed with 31 J/cm^2^ radiant exposure. For this, samples were light-cured for 20 s over the 6 mm surface with a 1550 mW/cm^2^ LED light-curing unit (LED Blue Star 3, Microdont, São Paulo, SP, Brazil). Irradiance was evaluated using a radiometer (RD-7, Ecel Indústria e Comércio Ltda, Ribeirão Preto, SP, Brazil) prior to the start of the experiment and after every 5 light activations to ensure the same conditions for every sample. The polymerization shrinkage induced stresses were analyzed by a specific software through the 50 Kgf load cell deformation. Data was recorded as force (in Newtons) × time (in seconds) in graphs and converted to MPa by dividing the force results by the area of transversal section of the specimens (12 mm^2^).

### Young's modulus evaluations

For Young's modulus test, a three-point bending test was used. Seven specimens of each resin were made through the insertion of the composite into a metallic matrix (2x2x25 mm=100 mm^3^ – ISO 4049) coated with a specific insulating gel (Gel tripla ação, KG Sorensen, Cotia, SP, Brazil).

The dimensions of specimens were standardized by positioning polyester strips (Kdent, Quimidrol, Joinvile, SC, Brazil) on both the upper and lower surfaces before light curing. The polymerization was performed on both the upper and bottom surfaces, in three points (left edge, right edge, and center), during 40 s for each one, according to ISO 4049 recommendations, for 240 s total and 372J/cm^2^.

The specimens were removed from the matrix and stored in distilled water at 37°C for 24 hours, in absence of light. Following, any excesses were removed with a 1200 grit silicon carbide paper (Buehler Ltd., Lake Bluff, IL, USA) adapted in a polishing machine. The Universal Testing Machine was then used. The specimens were adapted in a flexural test device with 3 metal barrels. Two of these (separated by 20 mm) were positioned on the bottom of the specimen. The third cylinder was positioned on the central upper surface, being responsible for the flexural stress. The force was applied through the superior articulated arm of the Universal Testing Machine at 0.5 mm/min crosshead speed until specimen fracture. Elasticity young's modulus was determined through the onboard Instron software.

### Statistical analyses

For all statistical analyses, 5% was adopted as the significance level (p<0.05). All data were evaluated for homogeneity through the Shapiro–Wilk test. For polymerization shrinkage stress, three-way ANOVA was used (time, composites and volume). For Young's modulus assessment, one-way ANOVA was used. All parametric tests mentioned above were followed by Tukey's test.

In addition, a linear regression analysis was performed considering Young's modulus and filler content, as well as considering Young's modulus and shrinkage stress.

## Results

Table 1 describes the shrinkage stress tests with 12 mm^3^ and 24 mm^3^, the comparison between the different resin composites, as well as the values for Young's modulus.

**Table 1:** Shrinkage stress (in MPa) for 12 and 24 mm3 increments, and Young's modulus (GPa) - Mean (standard deviation)

Time	20 s		300 s		
Group/Volume	12 mm^3^	24 mm^3^	12 mm^3^	24 mm^3^	Young's modulus
ADM	0.208 (0.02)^ADa^	0.233 (0.017)^Aa^	0.426 (0.026)^BCa^	0.508 (0.034)^ABb^	10.26 (1.38)^BE^
FBP	0.229 (0.029)^ABa^	0.288 (0.025)^ABa^	0.433 (0.035)^BCa^	0.493 (0.4)^ABa^	17.2 (1.08)^D^
FBF	0.283 (0.019)^BCa^	0.432 (0.027)^Db^	0.527 (0.036)^Da^	0.725 (0.054)^Db^	7.98 (0.32)^A^
SDR	0.199 (0.015)^Aa^	0.248 (0.023)^ABa^	0.386 (0.021)^ABa^	0.453 (0.037)^Aa^	8.62 (0.45)^AB^
TBF	0.171 (0.021)^Aa^	0.316 (0.027)^BCb^	0.328 (0.033)^Aa^	0.548 (0.023)^BCb^	12.39 (1)^C^
XB	0.315 (0.015)^Ca^	0.515 (0.033)^Eb^	0.525 (0.021)^Da^	0.77 (0.052)^DEb^	10.83 (0.68)^CE^
XF	0.214 (0.019)^ABa^	0.356 (0.04)^Cb^	0.384 (0.028)^ABa^	0.601 (0.04)^Cb^	21.6 (1.38)^F^
Z3F*	0.535(0.028)^Ea^	0.788 (0.066)^Fb^	0.880 (0.041)^Ea^	1.116 (0.034)^Fb^	8.3 (0.98)^A^
Z3XT*	0.272 (0.019)^BCDa^	0.524 (0.021)^Eb^	0.473 (0.018)^CDa^	0.831 (0.036)^Eb^	17.77 (1.69)^D^

* Conventional (non-bulk-fill) composites

Upper case letters mean statistically significant differences between rows in the same column (inter-groups), p≤.0.05

Lower case letters mean statistically significant difference between columns (intra-group) within the same row, regarding the different evaluated times (20 s - 12 mm3 versus 24 mm^3^; and 300 s - 12 mm^3^ versus 24 mm^3^), p≤.0.05

Considering 12 mm^3^ (Table 1), after 20 s, all bulk-fill composites, except FBF and XB, were similar. Only SDR and TBF generated significantly lower stress when compared to the conventional Z3XT. After 300 s, TBF, XF, and SDR generated the lowest stress values, followed by the other bulk-fill composites, which were similar to Z3XT. Z3F generated the highest stress values for all evaluated times.

Considering 24 mm^3^ specimens (Table 1), after 20 s, all bulk-fill composites, except XB, generated lower stress values than Z3XT. After 300 s, SDR, FBP and ADM generated the lowest stress values, followed by TBF and XF. Z3F generated the highest stress for all evaluated times.

After the volume increase, only ADM, FBP, and SDR generated similar values (20 s), regardless of the material volume (Table 1). After 300 s, SDR and FBP presented similar values regarding the different increment volumes.

**Figure 3:**
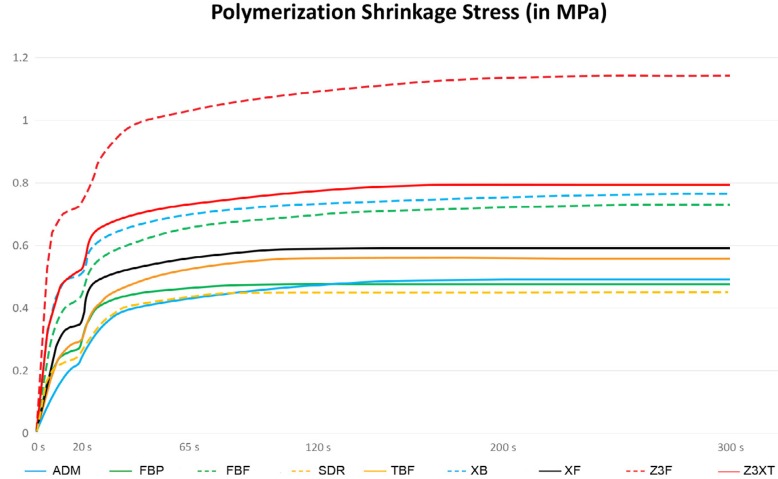
Stress development (in MPa) among the different composites (24 mm^3^)

Figure 3 shows the development of the shrinkage stress for the different composites. All bulk-fill composites showed smaller vertical lines when compared to their regular counterparts (Z3XT or Z3F). ADM showed the smallest vertical line, meaning that stress generation was slower.

Considering Young's modulus, the flowable composites (FBF, Z3F, SDR, and XB) presented the lowest values, followed by some high-viscosity bulk-fill composites (ADM and TBF). It is important to note that ADM and TBF presented statistically similar values to XB. The high-viscosity composites FBP, Z3XT, and XF presented the highest elastic modulus among the evaluated composites.

**Figure 4:**
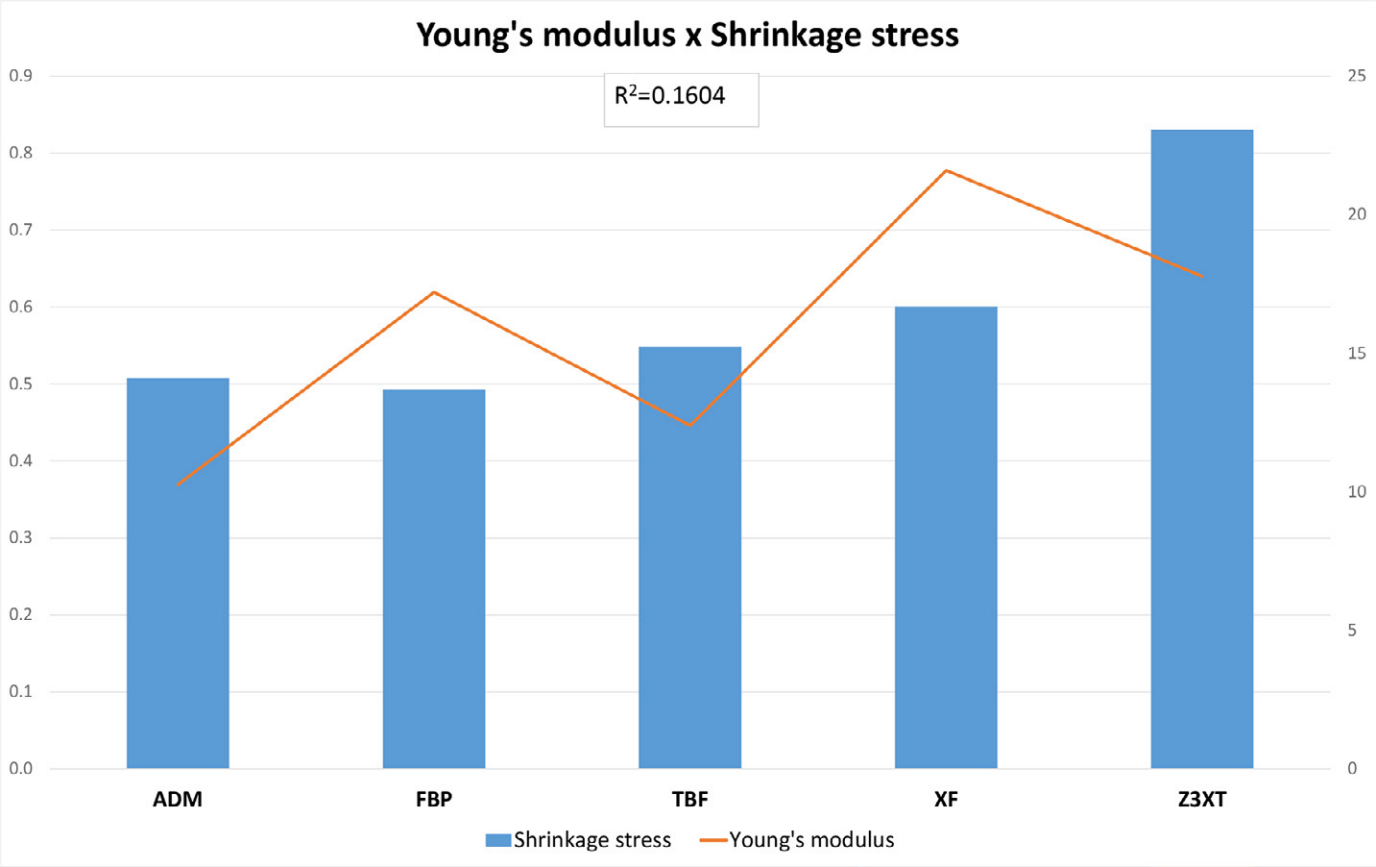
Young's modulus (GPa) x Shrinkage Stress (Mpa) for high-viscosity composites

**Figure 5:**
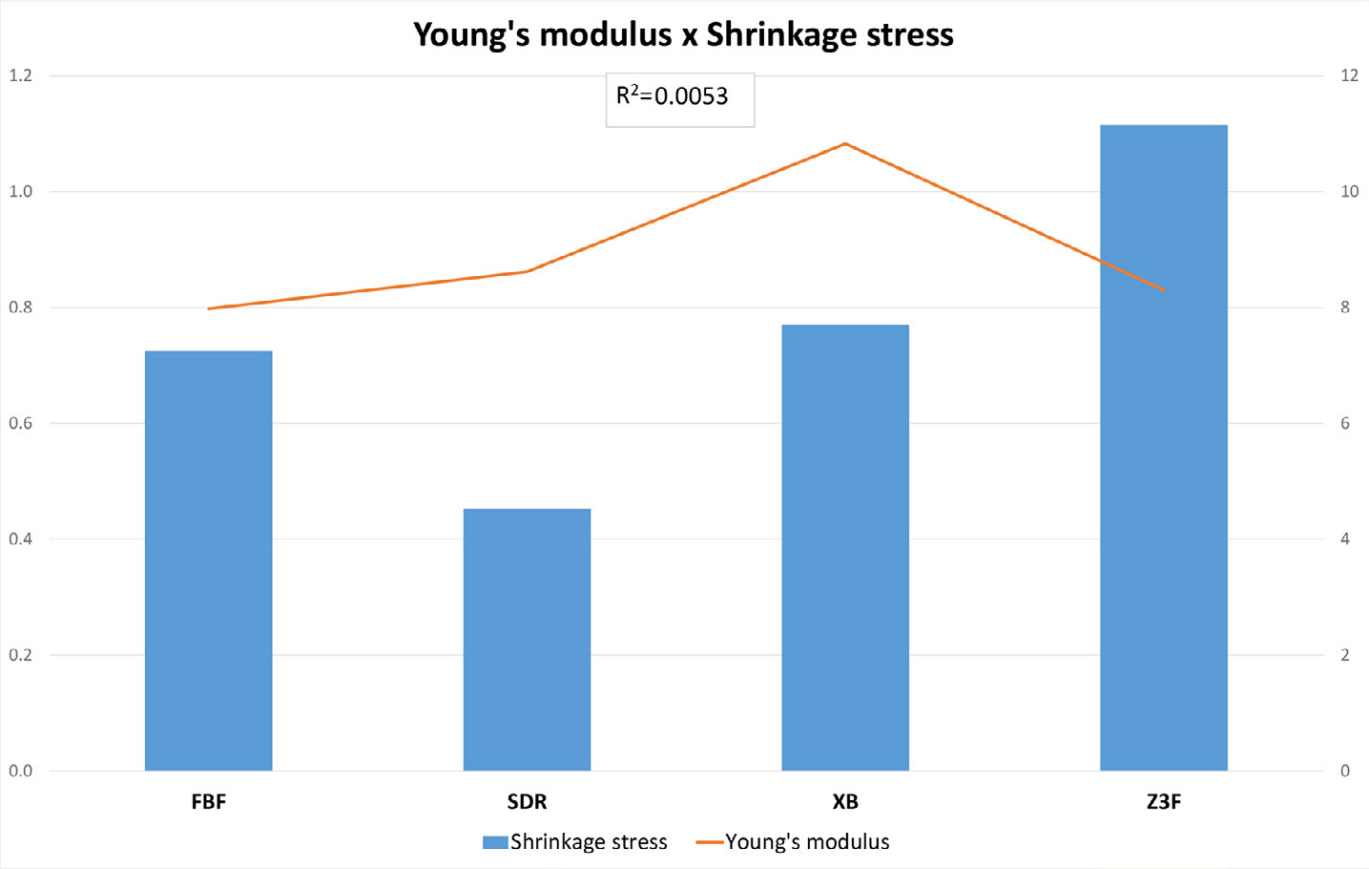
Young's modulus (GPa) x Shrinkage Stress (MPa) for low-viscosity composites

Considering the linear regression between Young's modulus and filler content, a lack of correlation for high-viscosity composites (R^2^=0.0636) and a strong correlation for low-viscosity composites (R^2^=0.9756) was observed. Another linear regression analysis was performed considering Young's modulus and shrinkage stress (Figures 4 and 5), and no correlation was observed for any of the composite groups.

## Discussion

Polymerization stress generated by the inherent shrinkage of composites during light curing has been the subject of several researches for a long time,^5^ ^,^ ^28^ since stress values that exceed adhesive resistance can lead to the formation of gaps in the interface.^5^ ^,^ ^29^ ^,^ ^30^ Therefore, the ideal composite should generate the lowest shrinkage stress possible while ensuring a better seal.^31^

To allow the insertion of larger increments, the molecular basis of bulk-fill composites was modified by the incorporation of stress relievers and monomers with higher molecular weight (low molecular weight monomers promote a higher number of double bonds *per* unit of weight, allowing a higher degree of conversion, but also leading to higher shrinkage and shrinkage stress).^5^ ^,^ ^16^ ^,^ ^18^ ^,^ ^20^ ^–^ ^23^ One may question the organic and inorganic matrixes of these composites since “conventional” and bulk-fill composites sometimes share similar composition. Nevertheless, manufacturers usually do not report the proportion between the different monomers, neither the filler content or their proprietary formulations.^16^ ^,^ ^20^ ^,^ ^22^ ^,^ ^28^

Similarly, differences in filler content (e.g., when comparing high- and low-viscosity composites) may be critical in volumetric shrinkage (higher stress due to a higher amount of organic content and lower filler content is expected in low-viscosity composites). Nevertheless, a lower Young's modulus may allow stress dissipation during the polymerization process, thus reducing the stress in bigger increments.^10^ ^,^ ^25^ ^,^ ^26^ ^,^ ^32^ ^,^ ^33^

Considering high-viscosity composites with 12 mm^3^ of material after 300 s, TBF and XF generated lower stress values when compared to the control group (Z3XT). The other bulk-fill composites presented values similar to Z3XT, but also similar to TBF and XF (Table 1). For low-viscosity/flowable composites, SDR generated the lowest stress values, followed by FBF and XB. The low-viscosity control group (Z3F) generated the highest shrinkage stress.

In general, high-viscosity bulk-fill composites generated lower shrinkage than low-viscosity bulk-fill composites as stated by other authors.^5^ ^,^ ^8^ ^,^ ^32^ The only exception was SDR, which generated similar stress when compared to high-viscosity bulk-fill composites despite being flowable. Such results can be explained by the presence of a modified UDMA (monomer with high molecular weight – 849 g/mol) which was stated to reduce shrinkage and, consequently, shrinkage stress.^34^ It is interesting to note that all bulk-fill composites (high- and low- viscosity) generated similar or lower stress values when compared to the high-viscosity control (Z3XT).

Given that stress depends on the composite volume,^29^ testing how the volume changes the impacts caused on shrinkage stress is important.^35^ Increased volume (24 mm^3^) resulted in increased stress for the evaluated composites. All bulk-fill composites with 24 mm^3^ generated lower or similar (XB) shrinkage stress when compared to Z3XT after 300 s (Table 1). SDR, FBP and ADM generated the lowest stress while Z3F generated the highest stress among all tested composites.

In addition, after 300 s, SDR, FBP and ADM with 24 mm^3^, showed values similar to Z3XT with 12 mm^3^, and FBP and SDR generated similar values for both 12 and 24 mm^3^ (Table 1). Such results demonstrate a great capability of bulk-fill composites in dealing with the generation of shrinkage stress, even in big increments, as previously reported.^22^ ^,^ ^36^

FBP relies on monomers with higher molecular weight (AUDMA, UDMA and 1, 12-dodecane-DMA), associated with a relatively higher filler content (76.5%) when compared to low-viscosity composites, to reduce polymerization shrinkage. The effect of monomers with higher molecular weight can be also observed in FBF, which substituted TEGDMA (286 g/mol) for UDMA and, despite presenting the same filler content as Z3F (~65% in weight), generated lower stress values. The same was observed for Xtra Base since the association between UDMA and increased filler content (75%) contributed for a lower shrinkage stress when compared to the conventional flowable composite.

Considering ADM, it relies on a new organic matrix (Ormocer), which seems to be more flexible (as observed in Young's modulus test), even with higher filler content (84% according to the manufacturer), probably resulting in a material with lower polymerization shrinkage and shrinkage stress.

It is important to note how the increase in volume affected the different classes of composites. Z3XT showed the biggest proportional increase in shrinkage stress. Considering high-viscosity bulk-fill composites, the lower volumetric shrinkage might have prevented a bigger increase in stress generation. In addition to showing stress values comparable with other bulk-fill composites, XF and TBF showed a big increase in stress. For XF, the higher filler content (86% in weight) probably reduces the polymerization shrinkage, resulting in stress values similar to other bulk-fill composites. Nevertheless, the higher Young's modulus (21.6±1.38 GPa) (Table 1), also reported in the literature,^37^ might have impacted the polymerization stress for XF (24 mm^3^) due to a sooner development of the composite viscosity during light curing, leading to a faster stress development when compared to some other high-viscosity bulk-fill composites (Table 1 and Figure 3). For TBF, the incorporation of 24% prepolymerized fillers increased the amount of filler content (80%), but it still might not be capable of reducing polymerization shrinkage. Regardless, the relatively high filler content – when compared to low-viscosity composites – combined to the inclusion of a monomer with lower viscosity and higher molecular weight (UDMA), resulted in a more flexible polymer (as observed in Young's modulus test) and reduced stress.

Young's modulus can contribute to a better stress distribution when the volumetric shrinkages of composites are similar. It can be noted that all tested flowable composites showed similar Young's modulus. The lower Young's modulus for flowable composites may explain why they showed slightly better stress distribution after the increase in the increment volume when compared to Z3XT (Table 1). The higher Young's modulus in high-viscosity bulk-fill composites can be compensated with a lower polymerization shrinkage, helping to lower stress generation as observed in this study. Interestingly, ADM (10.26±1.38 GPa) and TBF (12.39±1.00 GPa) presented lower Young's modulus when compared to other high-viscosity composites. For ADM, the ORMOCER-based organic matrix is responsible for a more flexible polymer, despite the high filler content. For TBF, the incorporation of 24% prepolymerized fillers increases the filler content without increasing the elastic modulus, as observed by other authors.^37^ ^,^ ^38^

Considering shrinkage stress development, a rapid increase during the first 10 s of light curing (20 s total) can be observed, followed by a slower increase until the LED light is turned off. The fast subsequent cooling of the composite might be responsible for a second shrinkage peak, as reported by other authors.^22^ ^,^ ^34^ ^,^ ^39^ Shrinkage stress development seems to be slower in bulk-fill composites when compared to conventional resins. This can be observed in Figure 3, in which stress development in bulk-fill composites took longer when compared to their regular counterparts. This is especially true when comparing bulk-fill composites within the same viscosity classification (i.e., high-viscosity bulk-fill composites with lower elastic modulus: ADM and TBF, showed slower stress development). This is important because a slower stress generation allows a better stress distribution and may contribute to the bonding integrity, since the material has more time to accommodate the shrinkage stress before the elastic modulus (composite stiffness) starts to increase.^40^

In addition, the stress curve is flat for all composites after 200 s, showing that most of the shrinkage develops during the initial minutes. This explains the current option for assessing shrinkage stress up to 5 minutes instead of several hours, as also observed by other authors.^7^ ^,^ ^32^ ^,^ ^39^

The authors of this study performed correlation tests between the elastic modulus and filler content. No correlation was observed for high-viscosity composites, but a strong correlation was observed for low-viscosity resins as reported by other authors.^39^ ^,^ ^40^ The low correlation between high-viscosity composites may have occurred because ADM and TBF present a relatively lower elastic modulus when compared to their filler content, as previously discussed.

In addition, no correlation was observed between shrinkage stress and elastic modulus for any of the composite groups (Figures 4 and 5). These results corroborate other authors.^34^ This can be explained by the high volumetric shrinkage of Z3XT and the use of monomers with higher molecular weight in FBP. Such results demonstrate the fundamental role of volumetric shrinkage on the generation of shrinkage stress.^34^ ^,^ ^40^ This statement supports the results of this study, since all flowable composites (with lower filler content) are expected to present higher shrinkage and generate higher shrinkage stress.^32^ The SDR group is an outlier as already discussed and as previously reported.^34^

Although having benefits that may reflect in easier and faster cavity restorations, bulk-fill composites still require further studies to assess the influence of their properties on the long-term maintenance of internal and marginal adaptation. Assessing the interaction between bulk-fill composites and tooth structure regarding adaptation, cusp deflection, among other factors, will also be important.

These results show, in general, a better behavior for bulk-fill composites regarding the generation of shrinkage stresses, mainly when larger increments are used. Nevertheless, it is important to note that despite being classified as bulk-fill resin composites, the different tested materials can show very different behavior, not only regarding the different classifications (low- and high-viscosity) as would be expected. Further tests are advised to clarify the best indication for each composite to clinicians. In addition, bulk-fill composites and regular composites also showed very different properties as previously discussed and, thus, the initial hypothesis was accepted.

## Conclusion

Considering the limitations of this study, it was possible to conclude that bulk-fill composites present very heterogeneous behavior, which is related to their composition (monomers and filler content).

In addition, it can be concluded that:

Bulk-fill resin composites present equal to lower shrinkage stress generation when compared to conventional composites, mainly with bigger increments.

Bulk-fill composites show a wide range of elastic young's modulus values, but usually similar to “regular” composites.

Volumetric shrinkage seems to be more important than elastic modulus for polymerization stress development.
